# Response to correspondence on ‘Open-access clinic for arthralgia: patient flow and diagnostic pathways—a retrospective cohort study’ by Nielsen et al.

**DOI:** 10.1016/j.ero.2026.100176

**Published:** 2026-04-27

**Authors:** Elisabeth Lindrup Nielsen, Kresten Krarup Keller, Jesper Blegvad Nissen, Tove Mark Lorenzen, Rene Drage Østgård, Tue Wenzel Kragstrup, Line Thorndal Moll, Peter Vedsted, Stine Daugaard Pedersen

**Affiliations:** Department of Rheumatology, Medical Diagnostic Center, University Clinic for Innovative Patient Pathways, Regional Hospital Central Jutland, Silkeborg, Denmark; Department of Rheumatology*,* Aarhus University Hospital*,* Aarhus*,* Denmark; Department of Clinical Medicine*,* Health, Aarhus University*,* Aarhus*,* Denmark; Department of Rheumatology, Medical Diagnostic Center, University Clinic for Innovative Patient Pathways, Regional Hospital Central Jutland, Silkeborg, Denmark; Department of Rheumatology, Medical Diagnostic Center, University Clinic for Innovative Patient Pathways, Regional Hospital Central Jutland, Silkeborg, Denmark; Department of Internal Medicine, Gødstrup Regional Hospital, Gødstrup, Denmark; Department of Rheumatology, Medical Diagnostic Center, University Clinic for Innovative Patient Pathways, Regional Hospital Central Jutland, Silkeborg, Denmark; Department of Biomedicine, Aarhus University, Denmark; Department of Molecular Medicine, Aarhus University Hospital, Denmark; Department of Rheumatology, Medical Diagnostic Center, University Clinic for Innovative Patient Pathways, Regional Hospital Central Jutland, Silkeborg, Denmark; Department of Clinical Medicine, Health, Aarhus University, Aarhus, Denmark; Research Unit for General Practice in Aarhus, Vennelyst Boulevard, Aarhus, Denmark; Medical Diagnostic Center, University Clinic for Innovative Patient Pathways, Regional Hospital Central Jutland, Silkeborg, Denmark; Department of Rheumatology, Medical Diagnostic Center, University Clinic for Innovative Patient Pathways, Regional Hospital Central Jutland, Silkeborg, Denmark

Response

We thank van Mulligen and van der Helm-van Mil for their important and thoughtful correspondence. A common and precise terminology on aspects of early arthritis recognition pathways is very much needed.

In their correspondence, the authors highlight that terminology related to early arthritis recognition clinics (EARCs) is not yet fully standardised across centres. It can be due to organisational structures and that referral pathways differ depending on local healthcare systems and the historical development of clinical services. In our setting, the open-access arthralgia clinic (OAC) was designed as an intermediate triage step between general practice and the EARC, offering rapid rheumatological assessment of patients with peripheral arthralgia without clear clinical arthritis. In contrast, the EARC serves as the setting for comprehensive diagnostic evaluation and treatment initiation for patients suspected of inflammatory arthritis, also referred to as the ordinary outpatient clinic for rheumatology. Although our OAC shares some features with what other groups describe as an EARC, the substantially lower proportion of patients eventually diagnosed with arthritis in our cohort suggests that the 2 settings were not equivalent and reflect differences in referral pathways, patient selection, and healthcare organisation.

We acknowledge that we must agree on the terminology used to describe similar clinical functions. As more initiatives aiming to facilitate early detection of inflammatory arthritis are implemented internationally, greater clarity and alignment in terminology could indeed enhance comparability between studies and clinical models. It would be relevant to discuss this in an international setting and agree on this terminology. Our use of ‘open access clinic’ came from a wish to signal that the access was easy and fast. However, it could also be the definition of an EARC.

We also appreciate the authors’ comments regarding the potential role of ultrasound in triage settings. In our clinic, ultrasound formed part of the brief assessment to support clinical evaluation. However, we agree that the optimal role of imaging in early arthritis detection pathways remains an important area for future research. It is our duty always to develop the most efficient, safe, and cost-effective diagnostic pathway.

Again, we thank the authors for starting this important discussion on improving early arthritis recognition. Another issue we have included in the paper is the terminology on milestones and time intervals.

## CRediT authorship contribution statement

**Elisabeth Lindrup Nielsen:** Writing – original draft, Writing – review & editing. **Kresten Krarup Keller:** Writing – review & editing. **Jesper Blegvad Nissen:** Writing – review & editing. **Tove Mark Lorenzen:** Writing – review & editing. **Rene Drage Østgård:** Writing – review & editing. **Tue Wenzel Kragstrup:** Writing – review & editing. **Line Thorndal Moll:** Writing – review & editing. **Peter Vedsted:** Writing – review & editing. **Stine Daugaard Pedersen:** Writing – review & editing.

